# Integrative analysis of circRNAs, miRNAs, and mRNAs profiles to reveal ceRNAs networks in chicken intramuscular and abdominal adipogenesis

**DOI:** 10.1186/s12864-020-07000-3

**Published:** 2020-08-26

**Authors:** Meng Zhang, Yu Han, Yanhui Zhai, Xiangfei Ma, Xinglan An, Sheng Zhang, Ziyi Li

**Affiliations:** 1 Key Laboratory of Organ Regeneration and Transplantation of Ministry of Education, First Hospital, Jilin University, Changchun, 130021 Jilin China; 2 College of Veterinary Medicine, Jilin University, Changchun, 130021 Jilin China; 3 College of Animal Science and Veterinary Medicine, Henan Agricultural University, Zhengzhou, 450002 Henan China

**Keywords:** Chicken, circRNAs, ceRNA, Intramuscular fat, Abdominal fat, Adipogenic differentiation

## Abstract

**Background:**

Tissue-specific fat deposition is regulated by a series of complex regulatory mechanisms. Reports indicate that epigenetic regulators, such as circular RNAs (circRNAs), are crucial in diseases progression, animal development, metabolism, and adipogenesis. In this study, to assess the functional roles of circRNAs in adipogenesis and tissue-specific fat deposition, we comprehensively analyzed the Ribo-Zero RNA-Seq and miRNAs data during chicken intramuscular and abdominal adipogenic differentiation.

**Results:**

circRNAs and miRNAs profiles during chicken adipogenic differentiation were found in adipocytes derived from various adipose tissues. It was also discovered that high levels of downregulated miRNAs potentially promote adipogenesis by activating their target genes which are associated with fatty acid metabolism and adipogenic differentiation. Through analysis of the correlation between the expression levels of circRNAs and adipogenic genes, as well as the dynamic expression patterns of circRNAs during adipogenic differentiation, several candidate circRNAs were identified. Moreover, competing endogenous RNA (ceRNAs) networks were constructed during chicken intramuscular and abdominal adipogenesis by combining miRNAs with mRNAs data. Several candidate circRNAs potentially influence adipogenesis by regulating miRNAs via PPAR and fatty acid metabolism-related pathways were identified, such as circLCLAT1, circFNDC3AL, circCLEC19A and circARMH1.

**Conclusion:**

In conclusion, our findings reveal that circRNAs and the circRNA-miRNAs-mRNAs-ceRNAs network may play important roles in chicken adipocytes differentiation and tissue-specific fat deposition.

## Background

Different resources and organizational microenvironment dictate various physiological and biochemical characteristics of adipose tissues [1–3]. Since preadipocytes derived from various tissues exhibit distinct proliferation speed and adipogenic potential, it is believed that transcriptional and post-transcriptional regulation mechanisms cause stage- and tissue-specific fat deposition in animals [4–6].

An increasing number of studies have shown that non-coding RNAs regulates various normal and pathological processes, such as microRNAs (miRNAs) [7–10] and long non-coding RNAs (lncRNAs) [11–13]. Although recent studies on the role of miRNAs and lncRNAs in adipogenesis have reached a mature stage, information on the functional roles of other classes of non-coding RNAs, such as circular RNAs (circRNAs) remains scanty. circRNAs are a class of non-coding RNAs generated by alternative splicing exhibiting weak protein-coding potential [14, 15]. Increasing evidence indicates that circRNAs promote various biological processes, such as tummorigenesis [16–18], animal development [19, 20] and cell differentiation [21, 22]. circHECTD1 promotes gastric cancer progression by targeting miR-1256 through activation of the β-catenin/c-Myc pathway [23]. circ-ZNF609, when translated to a functional petide, regulates myogenesis in humans and mice [24]. circArhgap5–2 plays a crucial role in adipogenesis and obesity [25]. However, circRNAs related to poultry adipogenesis and tissues-specific fat deposition remain poorly studied.

In this study, comprehensive circRNAs atlas during chicken adipogenic differentiation across various tissues-derived preadipocytes were provided. Future, we identified differentially expressed (DE) circRNAs, miRNAs and their dynamic expression patterns during adipocytes differentiation. Then, circRNAs and RNA-Seq data were combined to construct circRNAs-miRNAs-mRNAs ceRNA network involved in chicken adipogenesis. This study provides an in-depth understanding of epigenetic underlying regulation mechanisms of adipogenesis and tissues-specific fat deposition in birds.

## Results

### Overview of circRNAs data by Ribo-zero RNA-Seq in chicken preadipocytes and adipocytes

**Fig. 1:**
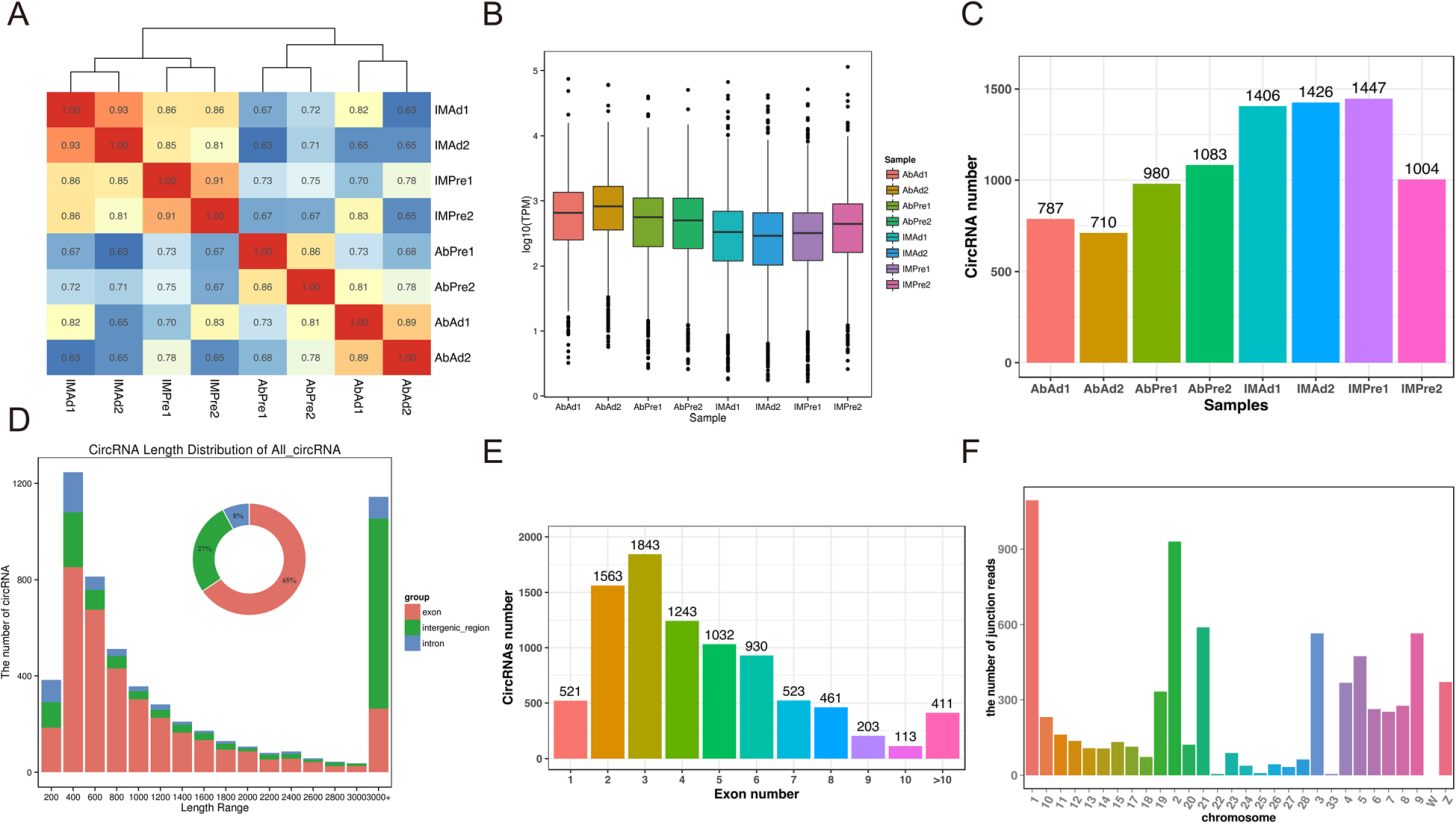
The characteristics of circRNAs in chicken adipocytes. **a** The correlations analysis among various samples. **b** The total expression levels of circRNAs in different groups. **c** The numbers of identified circRNAs in various samples. **d** The length and genome region distribution of all circRNAs. **e** The exon number distribution of circRNAs in chicken adipocytes. **f** The chromosome distribution of all circRNAs

**Fig. 2:**
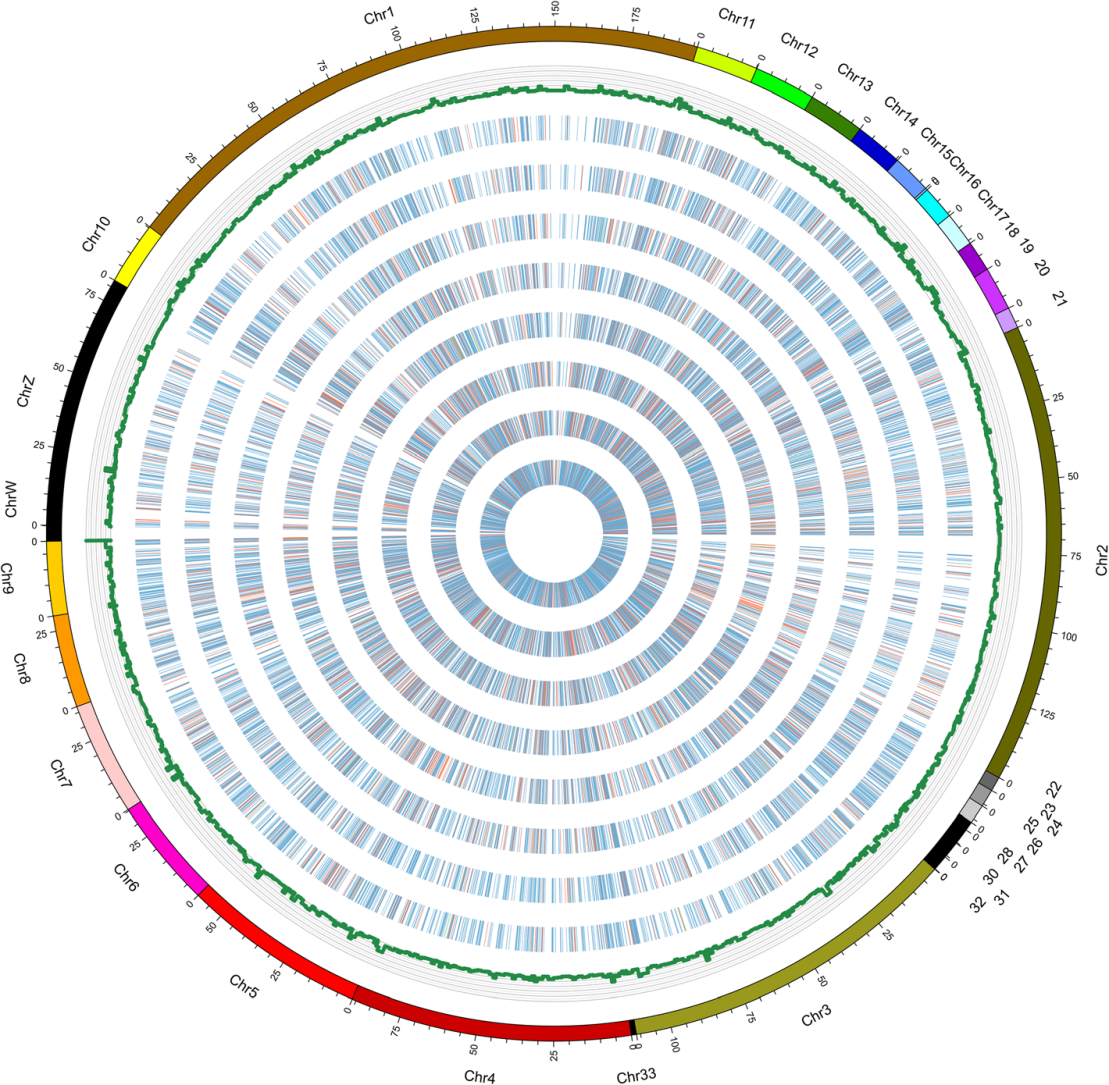
The circos plot of circRNAs in chicken adipocytes. The ring from the outer layer to the inner layer represent represents AbPre1, AbPre1, AbAd1, AbAd2, IMPre1, IMPre2, IMAd1and IMAd2 groups, respectively

Here, we analyzed 960 million raw paired-end (PE) reads generated by Ribo-Zero RNA-Seq data. After filtering with low-quality reads, we retained a total of 910 million clean reads. Unique clean reads of 88–94% were mapped to the chicken genome 5 (galGal5) (Table 1). It was found that circRNAs were distributed in most of the chicken chromosomes. The expression levels of circRNAs in different tissue-derived adipocytes from the adipose tissue were identified then analyzed to find a better understanding of the cirRNAs in chicken adipocytes. As shown in Fig. 1a, intramuscular preadipocytes (IM_Pre) and adipocytes (IM_Ad), abdominal preadipocytes (Ab_Pre), and adipocytes (Ab_Ad) were fell into two clusters, suggesting that the differences within the adipose tissues are potentially larger than that of adipocytes. Moreover, the Pearson correlation coefficient of circRNA expression between the cell samples within groups waved from 0.86 to 0.93, suggesting a reliable consistency of cell samples within groups. The bar plot showed that no apparent difference in global expression levels of circRNAs across different groups (Fig. 1b). To classify the length distribution of circRNAs, the resource of all circRNAs in different gene regions were compared, including exon, intergenic, and intron regions. Eventually, 787, 710, 980, 1083, 1406, 1426, 1447 and 1004 circRNAs were identified in eight groups respectively (Fig. 1c). Results showed that the majority of circRNAs (65%) were generated from exon regions, followed by intergenic regions (27%) and intron regions (8%) (Fig. 1d). As shown in Fig. 1d, most of the intersect circRNAs (60%) shared from different software were 200–1000 nt in length. Moreover, a considerable number of circRNAs which longer than 3000 nt were identified in the present study (Fig. 1d). Besides, most of circRNAs (94%) were found more than two exons (Fig. 1e). The circRNAs were distributed across 31 chromosomes (chromosomes 1–28, 33, Z, and W) of chicken with chromosomes 1, 2, 3 and 21 carrying the largest share (Fig. 1f, Fig. 2).

**Table 1:** An overall review of the circRNAs data in the present study

BMK-ID	Total Reads	Mapped Reads	Uniq Map Reads	Reads Map to ‘+’	Reads Map to ‘-’	GC(%)	Q30(%)
AbAd1	75,595,006	75,557,008 (99.95%)	70,918,567 (93.81%)	37,002,982 (48.95%)	37,013,048 (48.96%)	46.67	90.8
AbAd2	126,110,450	125,959,112 (99.88%)	118,894,690 (94.28%)	61,664,929 (48.90%)	61,680,043 (48.91%)	46.03	91.1
AbPre1	114,650,344	114,569,360 (99.93%)	107,125,637 (93.44%)	55,851,419 (48.71%)	55,649,631 (48.54%)	46.85	90.61
AbPre2	119,804,518	119,721,318 (99.93%)	111,531,581 (93.09%)	58,436,387 (48.78%)	58,456,954 (48.79%)	47.1	91.15
IMAd1	100,975,170	100,800,650 (99.83%)	91,276,252 (90.39%)	49,182,680 (48.71%)	48,704,390 (48.23%)	48	91.27
IMAd2	107,859,100	107,660,296 (99.82%)	96,647,866 (89.61%)	52,391,877 (48.57%)	52,116,610 (48.32%)	48.6	90.86
IMPre1	120,678,792	120,540,008 (99.88%)	111,158,225 (92.11%)	58,476,590 (48.46%)	58,500,070 (48.48%)	48.8	90.49
IMPre2	96,725,572	96,635,868 (99.91%)	85,825,333 (88.73%)	47,124,244 (48.72%)	47,116,981 (48.71%)	48.85	91.49

### Identification of differentially expressed (DE) circRNAs during adipogenic differentiation across various groups

**Fig. 3:**
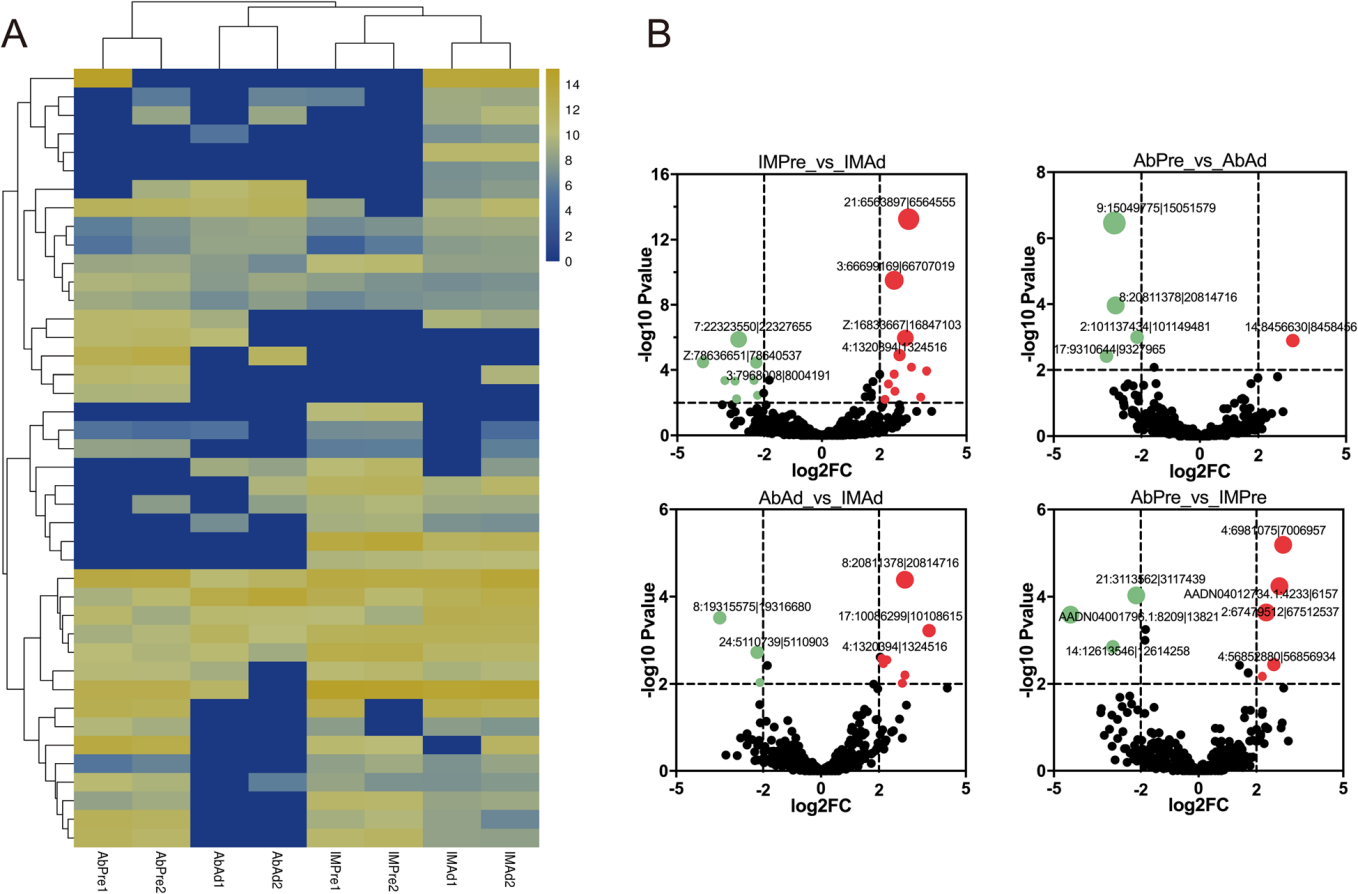
The identification of DE circRNAs across various groups. **a** Cluster analysis of DE circRNAs in different groups. **b** Volcano plots of DE circRNAs across various comparisons

To explore functional roles of circRNAs in tissues-specific adipogenic differentiation, we analyzed and compared the expression levels of circRNAs between different groups. Eventually, we identified 17, 14, 20, and 9 differentially expressed (DE) circRNAs across different groups (IMPre_vs_IMAd, AbPre_vs_AbAd, AbPre_vs_IMPre and AbAd_vs_IMAb) (Fig. 3, Table 2). Most of DE circRNAs were specificially found in preadipocytes or adipocytes. In addition, several DE circRNAs showed different expression patterns during adipogenic differentiation between AbF and IMF groups (Table S1). Among them, 7:22323550|22,327,655 was significantly downregulated in mature adipocytes when compared with preadipocytes in both IMF and AbF groups. Z:78636651|78,640,537 (circCDKN2A) was considerably downregulated in AbAd group, while upregulated in the IMAd group. Additionally, 7:22754779|22,756,470 (circTNS1) was only found in intramuscular fat preadipocytes and adipocytes, 21:6563897|6,564,555 (circHSPG2), 3:66699169|66,707,019 (circSLC22A16), and 16:242878|309,387 (circKIFC1L) were specifically expressed in intramuscular adipocytes. 3:79378778|79,415,580 (circBCKDHB), 28:1095851|1,096,498 and 1:83410785|83,437,136 were specifically expressed in abdominal adipocytes.

**Table 2:** The statistics of DE circRNAs across various comparisons

Group	ALL_DE circRNAs	Up_regulated	down_regulated
AbAd_vs_IMAd	9	8	1
AbPre_vs_AbAd	14	2	12
AbPre_vs_IMPre	20	9	11
IMPre_vs_IMAd	17	10	7

### Functional characterization of DE circRNAs

**Fig. 4:**
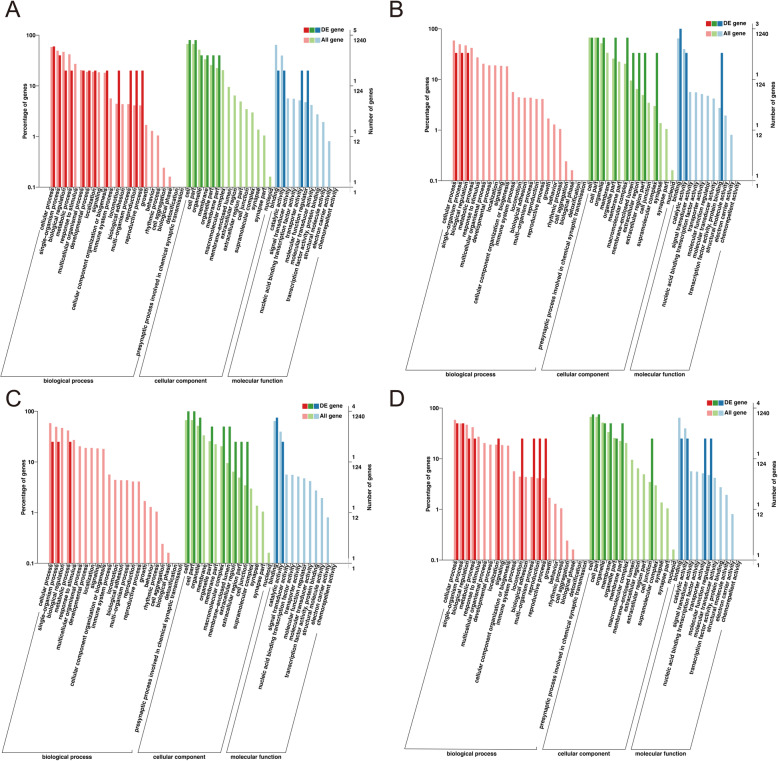
GO analysis of host genes of DE circRNAs across various groups. **a** IMAd vs IMPre. **b** AbAd vs AbPre. **c** AbAd vs IMAd. **d** IMPre vs AbPre

To investigate the potential functions of DE circRNAs, GO terms analysis of host genes of DE circRNAs were performed. We found that the molecular function (MF) of gene ontology (GO) analysis during adipogenesis were primarily enriched in lipid binding, phospholipase activity, and lipase activity, whereas cell component (CC) were mainly enriched in regulation of autophagy, glycerophospholipid biosynthetic process, phosphatidylinositol-mediated signaling and inositol lipid-mediated signaling (Fig. 4).

### Validation of circRNAs

**Fig. 5:**
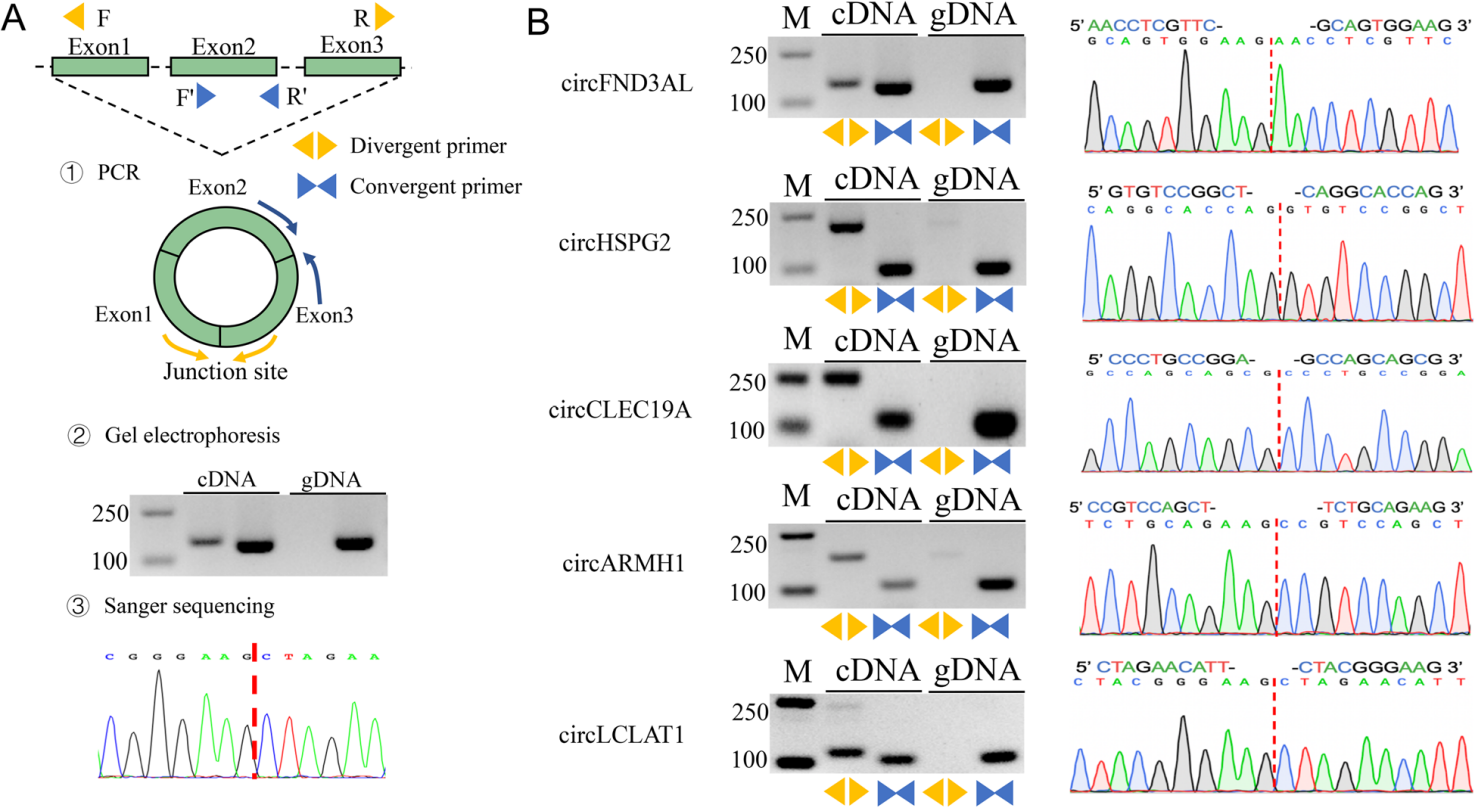
The validation of circRNAs in chicken adipocytes. **a** The validation flow of circRNAs in the present study. **b** The results of gel electrophoresis (left panel) and Sanger sequencing (right panel) of circRNAs. M, DL2000 DNA maker. The red dotted line represents junction sites

To confirm the circRNAs in chicken adipocytes, five DE circRNAs including circFNDC3AL, circHSPG2, circCLEC19A, circARMH1, and circLCLAT1 were randomly selected for validation using PCR and Sanger sequencing (Fig. 5a). Divergent and convergent primers were designed to amplify circRNAs back-spliced junction (BSJ) site and linear mRNAs. As showed in Fig. 5b, BSJ sites of circRNAs were amplified and validated by sanger sequencing. The amplified PCR products using convergent primers of circRNAs were distinct both in cDNA and gDNA samples, whereas the divergent primers only amplified the cDNA samples. PCR products amplified using the divergent primers were further identified through Sanger sequencing (Fig. 5b). The sequencing results corresponded to Ribo-zero RNA-Seq data, which suggested that the circRNAs data is reliable.

### Expression characteristics of miRNAs in chicken intramuscular and abdominal adipogenesis

**Fig. 6:**
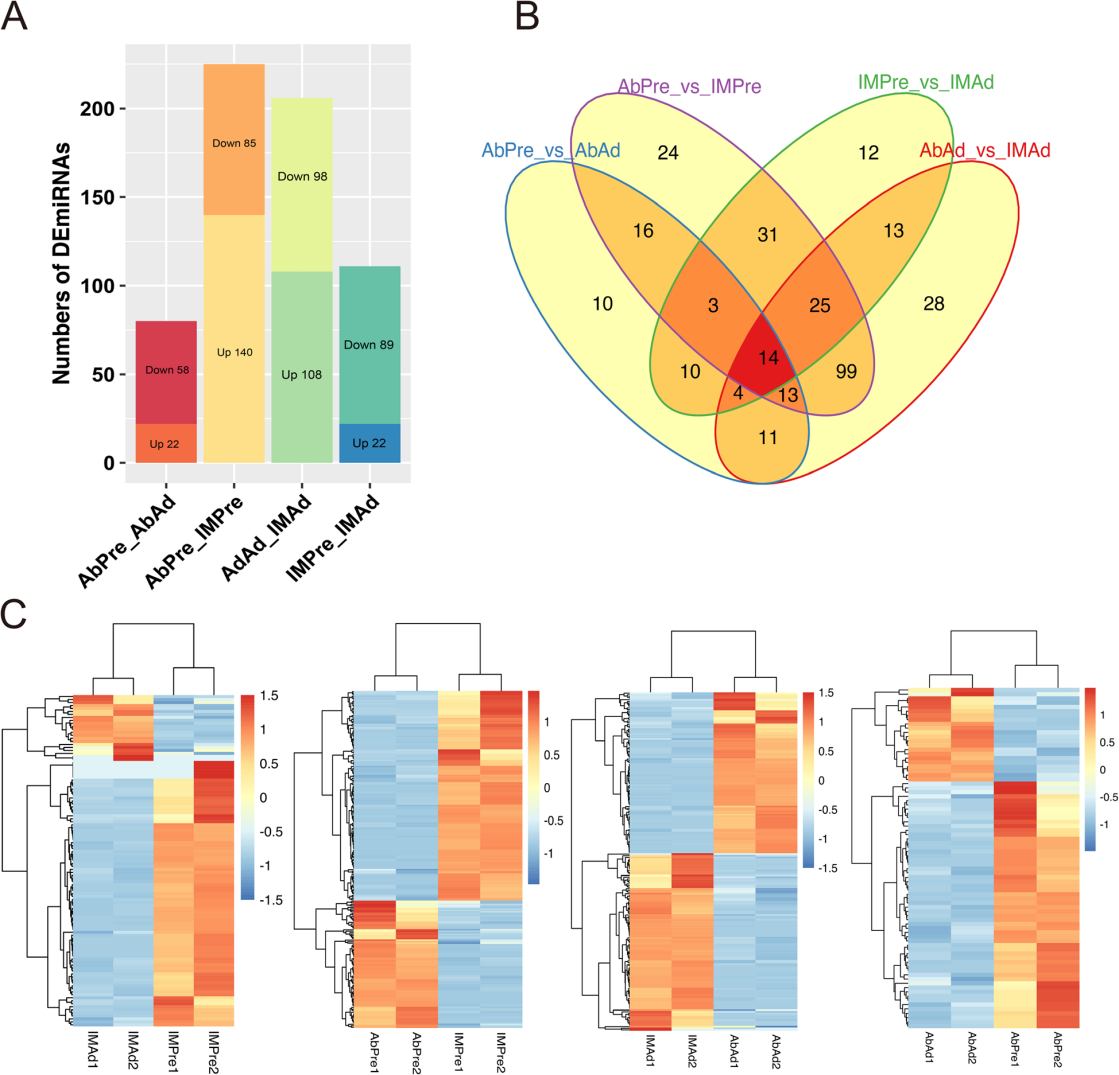
The characteristics of miRNAs during adipogenesis in different groups. **a** The numbers of DE miRNAs across various groups. **b** The Venn plot of DE miRNAs across various groups. **c** Heatmaps of DE miRNAs between different groups (IMAd vs IMPre, AbPre vs IMPre, IMAd vs AbAd, AbAd vs AbPre)

To comprehensively analyze the characteristics of miRNAs during tissue-specific adipogenesis, miRNAs expression levels were compared across various groups. A total of 80 DE miRNAs (58 upregulated and 22 downregulated) were identified between AbPre and AbAd groups, 225 DE miRNAs (85 downregulated and 140 DE miRNAs upregulated) between AbPre and IMPre groups, 206 DE miRNAs (98 downregulated and 108 upregulated) between AbAd and IMAd groups and 111 DE miRNAs (89 upregulated and 22 upregulated) between IMPre and IMAd groups respectively (Fig. 6a) (Table S2). Among them, 81 (66 up-regulated, 15 down-regulated) and 50 (34 up-regulated, 16 down-regulated) specific-DE miRNAs were identified in IM and Ab groups during adipogenic differentiation respectively (Fig. 6b). Moreover, it was observed that most of DE miRNAs were significantly downregulated in mature adipocytes, including miR-130b-5p, miR-148a-5p, miR-15c-5p, miR-16c-5p, miR-30a-3p (Fold change > 2, Q_value < 0.001). However, miR-146b-5p was significantly upregulated in mature adipocytes compared to preadipocytes (Fold change > 4, Q_value < 0.001). The heatmaps of DE miRNAs across various groups are displayed in Fig. 6c.

### Construction of circRNAs-miRNAs-mRNAs network

**Fig. 7:**
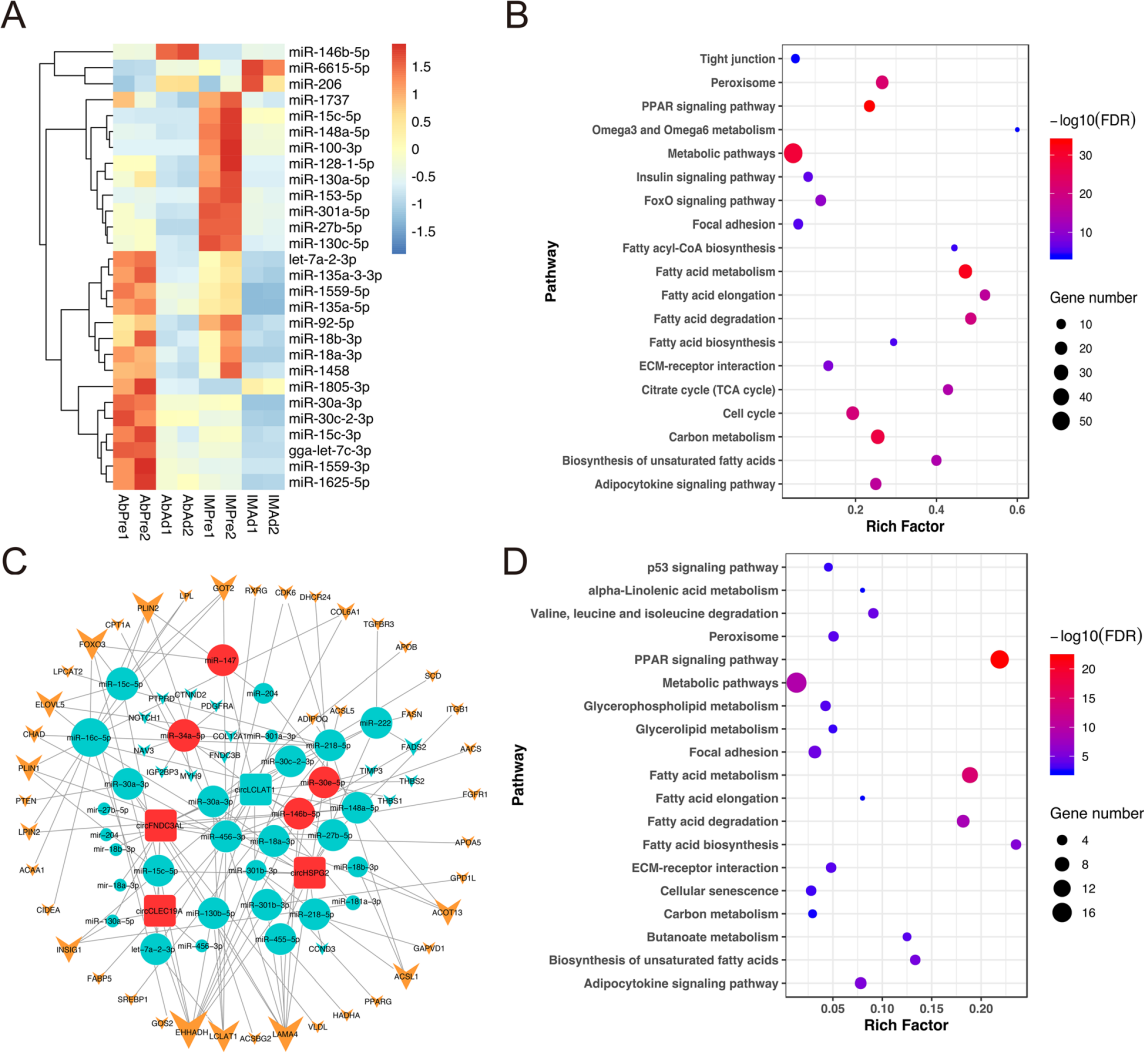
The construction of circRNAs-miRNAs-mRNAs ceRNAs networks during chicken adipogenesis. **a** Heatmap of 28 DE miRNAs across various groups. **b** The pathway enrichment analysis of target genes of miRNAs. **c** The circRNAs-media ceRNAs networks during chicken adipogenesis. The cube represents circRNAs, circles represent miRNAs and arrows represent genes. Sea-blue color represents a downregulated trend, while dark-red and brown-yellow color represents an upregulated trend. **d** The pathway enrichment analysis of downstream genes in ceRNAs networks

Notably, 31 DE miRNAs were shared between IM and Ab groups during adipogenesis, including miR-15c-5p, miR-206, miR-148a-5p, miR-128-1-5p, miR-30a-3p, miR-27b-5p, miR-92-5p and three novel miRNAs (not shown) (Fig. 7a). To further investigate the functional roles of miRNAs in chicken adipogenesis, the target genes of DE miRNAs were predicted through bioinformatics analyses. KEGG pathway analysis revealed that their target genes were highly enriched in the PPAR signaling pathway, metabolic pathways, fatty acid metabolism pathway (Fig. 7b). Further, through integrating analysis of DE cirRNAs, miRNAs and mRNAs data, we constructed a ceRNAs network during chicken adipogenesis (Fig. 7c). It was noted that circFNDC3AL, circHSPG2, circLCLAT1 and circCLEC19A were the hub cirRNAs of the ceRNAs network (Fig. 7c). Furthermore, KEGG pathway analysis showed that the ceRNAs network regulates target genes mainly via fatty acid metabolism, focal adhesion and ECM-receptor interaction pathways (Fig. 7d).

### Candidate circRNAs related to adipogenic differentiation

**Fig. 8:**
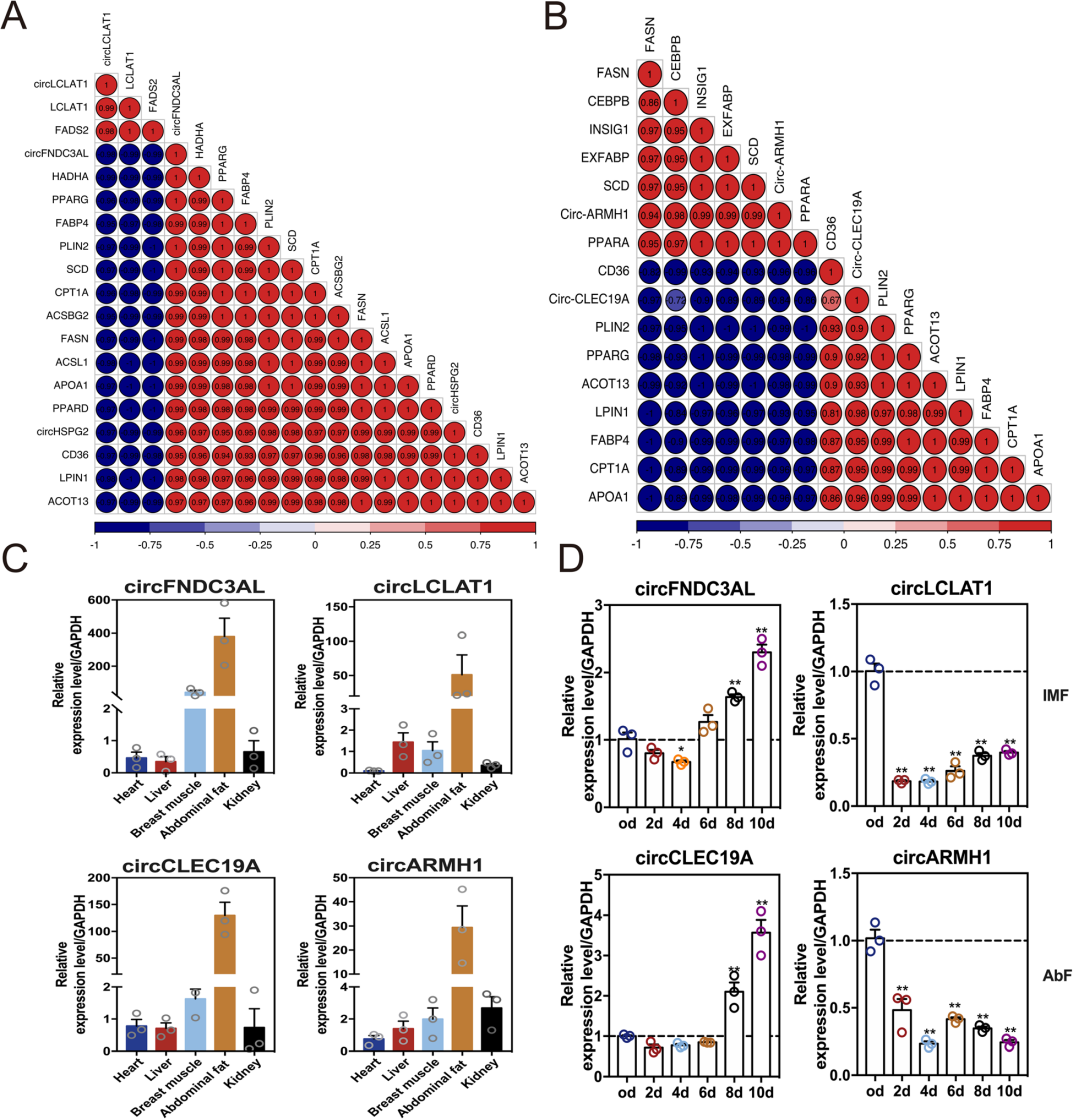
Candidate circRNAs associated with tissue-specific adipogenesis. **a** The Pearson correlations between the expression levels of circFNDC3AL, circHSPG2, circLCLAT1, (**b**) circARMH1, and circCLEC19A and genes associated with adipogenesis and lipid metabolism, respectively. **c** The relative expression levels of candidate circRNAs in five chicken tissues. **d** The dynamical expression patterns of candidate circRNAs during IM and Ab adipogenic differentiation, respectively (*n* = 3, Mean ± S.E.M). *p < 0.05, **p < 0.01

Further, correlation analysis between circRNAs and their target genes based on the ceRNAs network was performed to investigate the crucial roles of circRNAs in the adipogenesis of chicken. As shown in Fig. 8a, the expression levels of circFNDC3AL and circHSPG2 were significantly and positively related to PPAR signaling pathway genes in intramuscular adipogenesis (r > 0.90, *p* < 0.01), such as peroxisome proliferator-activated receptor γ (*PPARγ*), fatty acid binding protein 4 (*FABP4*), perilipin 2 (*PLIN2*), CD36 molecule (*CD36*). In contrast, the expression level of circLCLAT1 was significantly and negatively related to PPAR signaling pathway genes and positively related to lysocardiolipin acyltransferase 1 (*LCLAT1*) and fatty acid desaturase 2 (*FADS2*) (r > 0.95, *p* < 0.01) (Fig. 8a). Moreover, the expression levels of circARMH1 were significantly and negatively related to those of genes in abdominal adipocytes (r > 0.92, *p* < 0.01), while circCLEC19A was significantly and positively related to those of genes (r > 0.90, *p* < 0.01) (Fig. 8b). To further investigate the potential roles of circRNAs in the fat tissue-specific deposition, the expression of cirRNAs was analyzed in different tissues. Our results demonstrated that the expression of these four candidate circRNAs was highly expressed in the abdominal fat tissue of chicken (Fig. 8c). In addition, the dynamistic expression patterns of candidate circRNAs were detected during tissue-specific adipogenetic differentiation. Our results showed that the expression levels of circFNDC3AL were decreased after 4 days of induction, while dramatically upregulated in the late stage of intramuscular adipogenesis (Fig. 8d). The expression levels of circCLEC19A were significantly increased during the late stage of abdominal adipogenesis (Fig. 8 d). In contrast, the expression levels of circCLEC19A were dramatically decreased after 2 days’ of adipogenetic induction, while slightly increased in the late stage of intramuscular adipogenesis. In addition, the expression levels of circARMH1 were decreased during the abdominal adipogenesis (Fig. 8d). Interestingly, we noticed that chicken circLCLAT1 was highly conservative among humans, mice and pigs (Fig. S1), implying that circLCLAT1 potentially regulates animal adipogenesis or lipid metabolism.

### Validation of circRNA-miRNA-mRNAs ceRNA networks

**Fig. 9:**
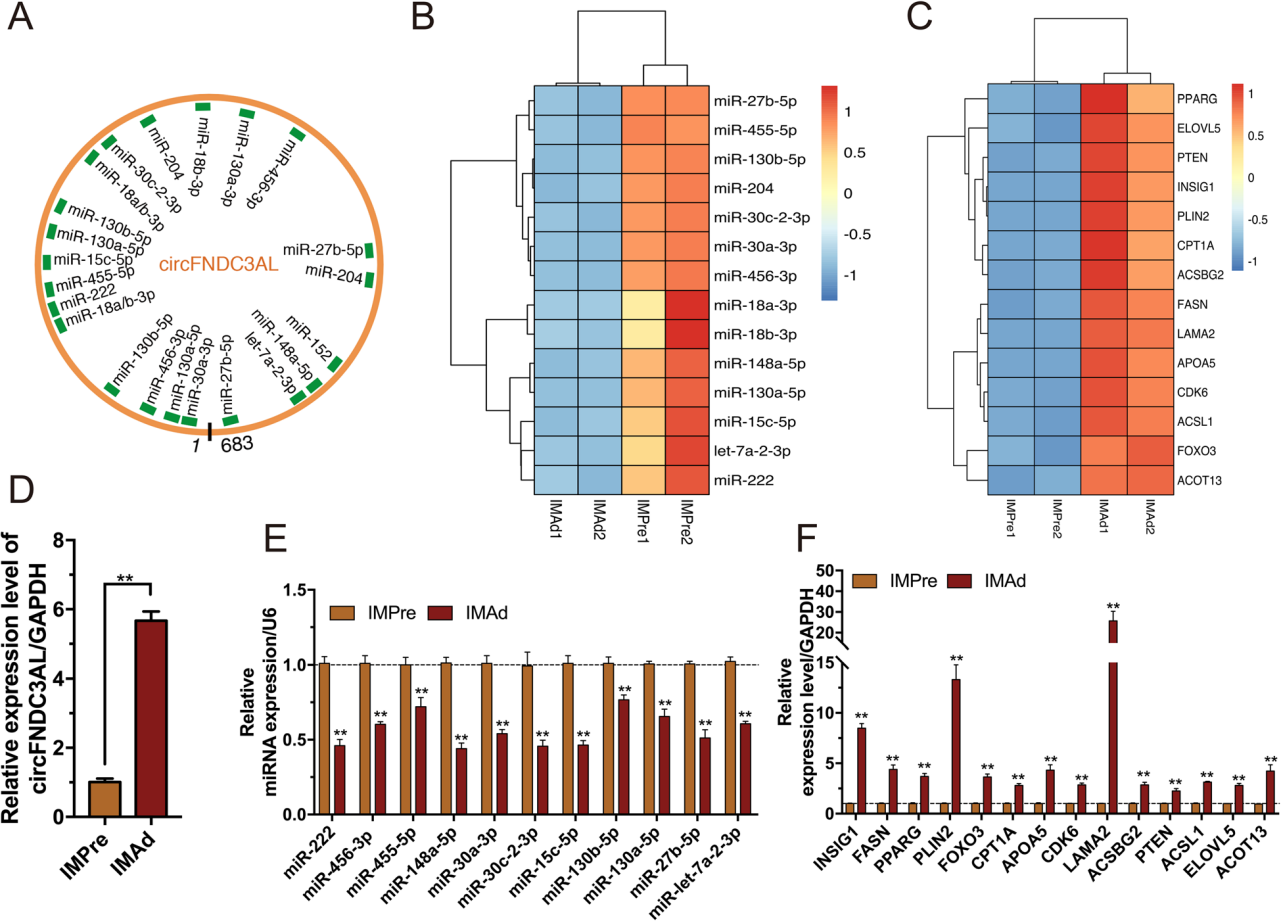
circFNDC3AL regulated chicken adipogenesis via the ceRNA pathway. **a** The miRNAs binding sites of circFNDC3AL. **b** The expression patterns of candidate miRNAs and (**c**) their target genes during intramuscular adipogenesis in RNA-Seq data. The expression levels of (**d**) circFNDC3AL, (**e**) miRNAs and (**f**) targets genes before and after intramuscular adipogenic differentiation. (n = 3, Mean ± S.E.M) *p < 0.05, **p < 0.01

**Fig. 10:**
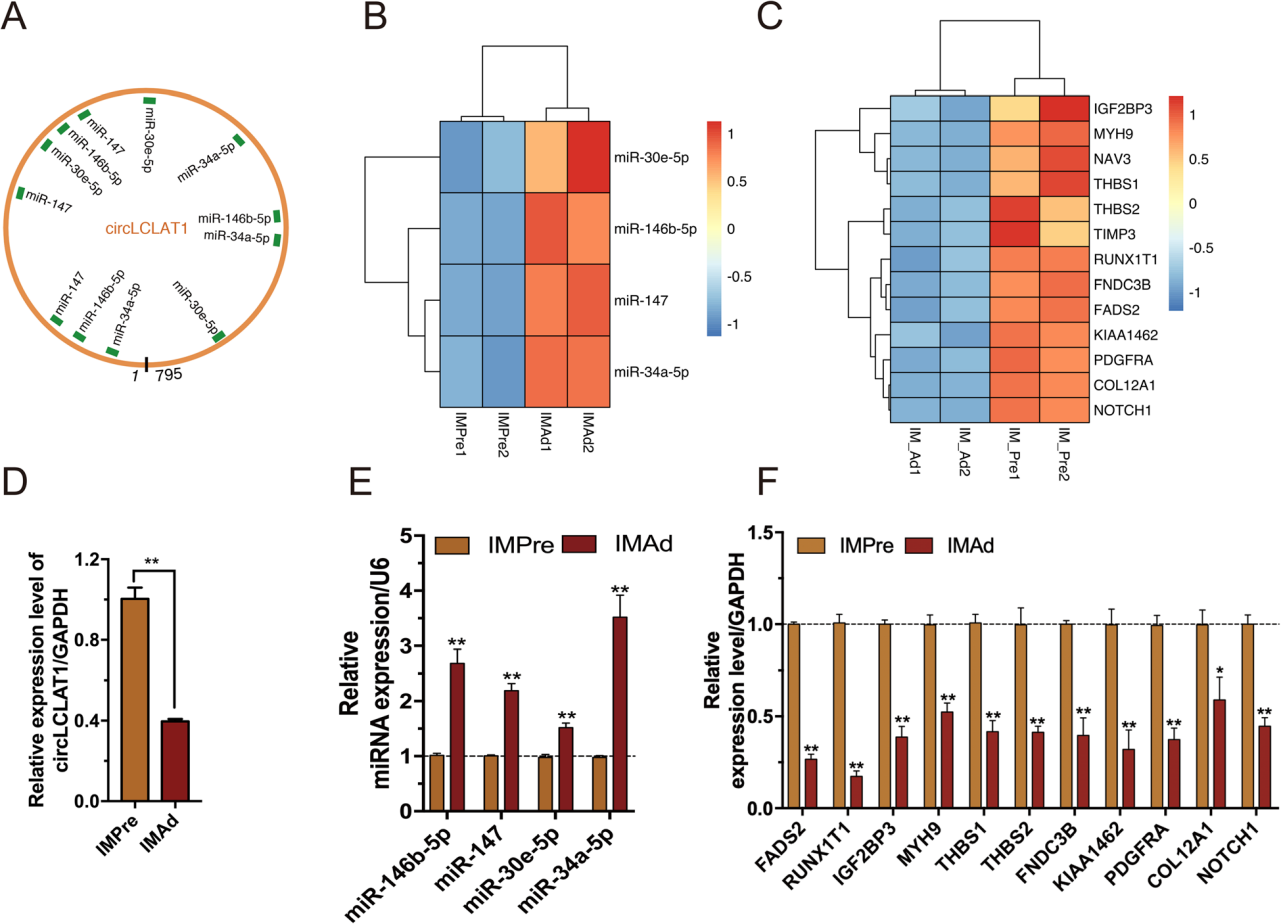
circLCLAT1 regulated chicken adipogenesis via the ceRNA pathway. **a** The miRNAs binding sites of circLCLAT1. **b** The expression patterns of candidate miRNAs and (**c**) their target genes during intramuscular adipogenesis in RNA-Seq data. The expression levels of (**d**) circLCLAT1, (**e**) miRNAs, and (**f**) targets genes before and after intramuscular adipogenic differentiation. (n = 3, Mean ± S.E.M) *p < 0.05, **p < 0.01

To verify the circRNA-miRNA-mRNAs regulation networks during intramuscular adipogenic differentiation, we analyzed their expression levels in intramuscular preadipocytes and mature adipocytes. Bioinformatics analysis revealed that cirFNDC3AL exhibit many MREs (miRNAs response elements), such as miR-130a-5p, miR-15c-5p, miR-18a/b-3p (Fig. 9a). Results from RNA-Seq and RT-qPCR showed that cirFNDC3AL and its downstream genes were significantly upregulated in differentiated-mature intramuscular adipocytes, compared to intramuscular preadipocytes (Fig. 9c, d, f). On the other hand, several adipogenic differentiation-related miRNAs were significantly downregulated in adipocytes, compared to intramuscular preadipocytes, including miR-222, miR-456-3p, miR-15C-5p, miR-130b-5p, miR-130a-5p (Fig. 9b, e). Additionally, it was worth noting that the target genes of circFNDC3AL were mainly associated with lipid metabolism and adipogenesis, such as *PPARG*, *PLIN2*, *CPT1A*, *INSIG1*. In addition, bioinformatic analysis and qRT-PCR results found that miR-146b-5p and miR-147 potentially regulate *RUNXT1* and *FADS2,* while miR-34a-5p and miR-30e-5p might regulate *PDGFRA*, *IGF2BP3*, *FNDC3B*, *MYH9* (Fig. S2). circLCLAT1 potentially influence chicken adipogenesis by sponging these miRNAs, thus regulating target genes. The findings suggested that circLCLAT1-miRNAs (miR-146b-5p, miR-147, miR-30e-5p, and miR-34a-5p)-mRNAs pathway showed opposite expression patterns during intramuscular adipogenic differentiation (Fig. 10). CircLCLAT1 and its downstream genes were significantly downregulated in differentiated-mature intramuscular adipocytes (Fig. 10c, d and f), while miR-146b-5p and miR-147 were significantly upregulated in mature adipocytes compared to preadipocytes (Fig. 10b, e).

## Discussion

Preadipocytes-derived from abdominal fat (AbF) have previously been reported to exhibit higher adipogenic differentiation potential than the ones derived from intramuscular fat (IMF) in chickens [26]. Adipogenesis is controlled by a series of complex regulatory mechanisms, including transcription level, post-transcription level, among others [27–29]. Noncoding RNAs have notable effects on adipogenesis and lipid metabolism [30, 31]. Several studies about noncoding RNAs on adipogenesis were geared towards mammals [11, 12, 30, 31]. However, information on circular RNAs (circRNAs) in poultry adipogenesis is limited.

In this work, we analyzed Ribo-zero RNA-Seq and corresponding miRNAs data during chicken tissue-specific adipogenic differentiation. Three software (CIRI2, CIRCexplore2 and find_circ) were used to identify circRNAs. Although fewer circRNAs were obtained with this way, it improved the accuracy rate of our findings. A total of 8843 circRNAs were identified in preadipocytes and adipocytes. This suggested that several circRNAs are expressed in chicken preadipocytes and adipocytes. Of note, the characteristics of circRNAs based on length, exon number, and genomic distribution in chicken were in line with previous reports [19, 32]. Previous studies revealed that the expression level of circRNAs was tissues- and stage-specific [33, 34]. Abdominal and intramuscular fat deposition resulted from a variety of complex molecular mechanisms. Notably, tissue-specific adipogenesis is potentially regulated by circRNAs [25]. In the present study, we identified DE circRNAs across various tissue and cell types. Also, previous reports identified several DE circRNAs in chicken, such as circCELC19A which was suggested to play functional roles in follicle development [32]. In addition, circFNDC3AL and circTNS1 were identified in embryonic muscle tissues of chicken [19].

Previously, we reported that miRNAs could be crucial regulators in chicken adipogenesis [8, 35]. Here, we systematically analyzed and compared the expression patterns of miRNAs during intramuscular and abdominal adipogenesis. It was revealed that several miRNAs were significantly downregulated in mature adipocytes. On the other hand, many genes associated with lipid metabolism and adipogenesis were upregulated during the adipogenic differentiation across various tissues, implying that the downregulated miRNAs potentially promote adipogenesis through their target genes in chicken.

Generally, it is believed that circRNAs could function as a molecular sponge of miRNAs thereby regulating their target genes [36]. By integrating the analysis of circRNAs, miRNAs, and mRNAs data, hub ceRNAs networks were constructed during chicken adipogenic differentiation. Notably, the hub genes in the ceRNAs network were enriched in fatty acid metabolism, PPAR signaling pathway, and p53 signaling pathway. Furthermore, the downstream genes in the ceRNA pathways were strongly related to candidate circRNAs in the present study (Fig. 7c and Fig. 8a), suggesting that these circRNAs might play functional roles during adipogenesis. RT-qPCR results of the circFNDC3AL-miRNAs-mRNAs pathway corroborated the results of bioinformatics analysis. Interestingly, fibronectin type III domain-containing protein 3B (*FNDC3B*) and 5 (*FNDC5*) regulate white fat browning and adipogenesis [37–40]. Besides, perlecan (HSPG2, Heparan Sulfate Proteoglycan 2) was found to regulate obesity and lipid deposition [41]. Thus, we speculated that circFNDC3AL and circHSPG2 potentially regulate chicken adipogenesis. However, in-depth studies on the functions of chicken circFNDC3AL and circHSPG2 on adipogenic differentiation are essential. Previous research reported that runt-related transcription factor 1 (*RUNX1T1*) [42, 43], myosin, heavy chain 9, non-muscle (*MYH9*), fatty acid desaturase 2 (*FADS2*), insulin-like growth factor 2 mRNA-binding protein 1 (*IGF2BP1*) were associated with adipogenesis and fat deposition [44–47]. We found that circLCLAT1 might influence adipogenic differentiation by regulating downstream genes (*RUNX1T1*, *FADS2, MYH9, IGF2BP3,* and *PDGFRA*) through four miRNAs (miR-34a-5p, miR-30e-5p, miR-146b-5p, and miR-147). Nonetheless, future in-depth studies should be undertaken to explore the molecular regulation mechanism of circFNDC3AL and circLCLAT1/miRNAs/mRNAs pathway in chicken adipogenesis.

## Conclusions

In summary, we comprehensively identified expression profiles of circRNAs in adipocytes derived from various adipose tissues in chicken. By integrating circRNAs, miRNAs, and mRNAs data, we constructed ceRNAs networks that regulated chicken adipogenesis. Over all, this study offers insights into poultry tissue-specific adipogenesis in poultry and reveals novel clues to studying circRNAs on adipogenic differentiation and fat deposition in poultry.

## Methods

### Tissues and cell samples collection

The experimental birds were obtained from the Avian Farm of Henan Agriculture University (Zhengzhou, Henan, China). Here, five types of tissues (heart, liver, breast muscle, abdominal, and kidney tissues) were collected and stored at − 80 °C until use. Under deep anesthesia, the birds were euthanized by intramuscular injection of pentobarbital (Sigma, St. Louis, MO, USA) (40 mg/kg). Intramuscular and abdominal preadipocytes were isolated from the breast muscle and abdominal adipose tissue of 2 weeks old-chicken following the method described by Zhang et al [8, 26]. Pollution-free treatment of animal carcasses was performed after the experiment. In vitro adipogenic differentiation model of chicken preadipocytes constructed according to the method by Zhang et al [26]. After 80–90% confluence, cells were exposed to the MDIO differentiation medium. Cell samples were harvested at 0, 2, 4, 6, 8, and 10 days after adipogenic induction.

### Data resources, annotation and quantification of circRNA

Ribo-Zero RNA-Seq and miRNAs data were downloaded from the NCBI database (No. PRJNA429489 and PRJNA453673). Four groups were included in the PRJNA429489 and PRJNA453673 program, including preadipocytes derived from chicken intramuscular fat (IM_Pre), abdominal fat (Ab_Pre) and differentiated-mature adipocytes (IM_Ad, Ab_Ad). Each group included two biological replicates. The paired-end (PE) reads were mapped to the *Gallusgallus* 5 genome (https://www.ncbi.nlm.nih.gov/genome/?term=chicken) using Bowtie2 (v2.2.9) with default parameters [48]. The output of Bowtie2 was scanned and analyzed by CIRI2 (v2.0.6) [49], find_circ (v1.0) [50] and CIRCexplore2 (v2.3.3) [51] with default parameters. The identification of circRNA was based on the intersection of the three software. TPM was applied to calculate the relative expression abundance of circRNA.

### Identification of differentially expressed (DE) circRNAs and host genes functional annotation

DE circRNAs were analyzed using the edgeR package [52], with the threshold of *p*-value < 0.05 and |fold change| ≥ 2. Host genes of circRNAs were used for predicting the potential functions of circRNAs. KOBAS 3.0 (http://kobas.cbi.pku.edu.cn/kobas3/?t=1) online software was used for Gene Ontology (GO) enrichment and Kyoto Encyclopedia of Genes and Genomes (KEGG) pathway analyses.

### The conservative analysis of circRNAs

CircRNAs sequences of humans and mice were downloaded from the circBase database (http://www.circbase.org), porcine circRNAs sequences were download from pigcircNet database (http://lnc.rnanet.org/circ/). The sequences were compared using Clustal Omega (https://www.ebi.ac.uk/Tools/msa/clustalo/) for multiple sequence alignment.

### Genomic DNA (gDNA) and total RNA extraction, cDNA synthesis and quantitative real-time PCR (qRT-PCR)

Genomic DNA (gDNA) and total RNA were extracted from tissues/cell samples using animal genomic DNA kit (Tiangen, China) and RNAiso plus (Takara, Dalian, China) following to the manufacturers’ instructions respectively. cDNA was synthesized with a PrimeScriptTM RT reagent Kit with gDNA Eraser (Takara, Dalian, China). Primers used for qRT-PCR were designed by primer3plus (Supplementary Table S3). qRT-PCR was performed with the SYBR® Premix Ex TaqTM II kit (Takara) on a LightCycler® 96 Real-Time PCR system. *GAPDH* and *U6* were used as the reference genes of circRNAs, mRNAs, and miRNAs, respectively. The 2^−ΔΔCt^ method was used to calculate gene expression levels.

### Validation of circRNAs

To validate the circRNAs related to chicken adipogenesis, PCR and Sanger sequencing assays were performed. Divergent primer was designed to amplify the junction sites of circRNA, whereas a convergent primer was designed to amplify the linear mRNA (Supplementary Table S3). Random primers were used for cDNA synthesis. Then, the cDNA of intramuscular and abdominal preadipocytes and adipocytes were mixed to a cDNA pool. The PCR products were sequenced by Sangon Biotech Co. Ltd. (Shanghai, China).

### The integrative analysis of circRNAs, miRNAs, and mRNAs

Since circRNAs can act as sponges of miRNA, the potential miRNAs binding sites in chicken adipogenesis were analyzed. The four groups of circRNA data including IMPre, IMAd, AbPre, and AbAd were analyzed during chicken adipogenesis were analyzed, and the corresponding four groups of miRNA data during adipogenesis were descripted and analyzed in our previous studies [35, 53]. miRNAs with |fold change| > 2 and FDR < 0.05 were regarded as differentially expressed miRNAs. TargetScan (http://www.targetscan.org/vert_72/), miRanda (http://www.microrna.org/microrna/home.do), and RNAhybrid software (https://bibiserv.cebitec.uni-bielefeld.de/rnahybrid) were used to predict the miRNA sites in mRNAs and circRNAs with default parameters, and investigate putative interactions between miRNAs and mRNAs or circRNAs. Furthermore, the target genes with opposite expression trends to miRNAs were regarded as candidate target genes. Based on the co-expression of DE circRNAs and miRNAs (|Pearson’s correlation coefficient| > 0.8 and *p* < 0.05), the circRNAs-miRNAs-mRNAs ceRNA networks were constructed and visualized using Cytoscape (version 3.5.0) (http://www.cytoscape.org/) [54].

### Statistical analysis

All statistical data were analyzed using SPSS 22.0 (SPSS Inc., Chicago, IL, USA). All results were presented as mean ± S.E.M, a two-tailed t-test to analyzed the data. *p* < 0.05 and ** *p* < 0.01 were considered significant and extremely significant respectively.

## Supplementary information

**Additional file 1: Table S1.** The list of differentially expressed (DE) circRNAs across various groups. **Table S2.** The list of differentially expressed (DE) miRNAs across various groups. **Table S3.** The primers used for qRT-PCR in the present study.

**Additional file 2 Figure S1.** circLCLAT1 is a sequence-conservative circRNA. (A) Genome locate of chicken circLCLAT1. (B) Multiple sequence alignment analysis of circLCLAT1 among species. (C) The evolutionary tree of circLCLAT1 among species.

**Additional file 3 Figure S2.** The binding sites of some upregulated miRNAs (eg. miR-146b-5p, miR-147, miR-34a-5p, miR-30e-5p) in the 3’UTRs of potential target genes.

## Data Availability

The sequencing data has been submitted to NCBI SRA under BioProject ID: PRJNA429489 and PRJNA453673.

## Abbreviations

IMF: Intramuscular fat
AbF: Abdominal fat
circRNAs: Circular RNAs
miRNAs: MicroRNAs
MREs: MiRNAs response element
DE: Differentially expressed
GO: Gene Ontology
KEGG: Kyoto Encyclopedia of Genes and Genomes
BSJ: Back-spliced junction
FNDC3AL: Fibronectin type III domain containing 3A-like
FNDC3B: Fibronectin type III domain-containing protein 3
FNDC5: Fibronectin type III domain-containing protein 5
TNS1: Tensin 1
HSPG2: Heparan sulfate proteoglycan 2
CLEC19A: C-type lectin domain containing 19A
ARMH1: Armadillo like helical domain containing 1
LCLAT1: Lysocardiolipin acyltransferase 1
RUNX1T1: Runt-related transcription factor 1
MYH9: Myosin, heavy chain 9, non-muscle
FADS2: Fatty acid desaturase 2
IGF2BP1: Insulin-like growth factor 2 mRNA-binding protein 1
PPARγ: Peroxisome proliferator-activated receptor γ
FABP4: Fatty acid binding protein 4
PLIN2: Perilipin 2
CD36: CD36 molecule
