# Supplementary figures and images for: Integrative analysis of circRNAs, miRNAs, and mRNAs profiles to reveal ceRNAs networks in chicken intramuscular and abdominal adipogenesis
Source: BMC Genomics. 2020 Aug 26;21:594. doi: 10.1186/s12864-020-07000-3 (PMC7450580; doi:10.1186/s12864-020-07000-3)

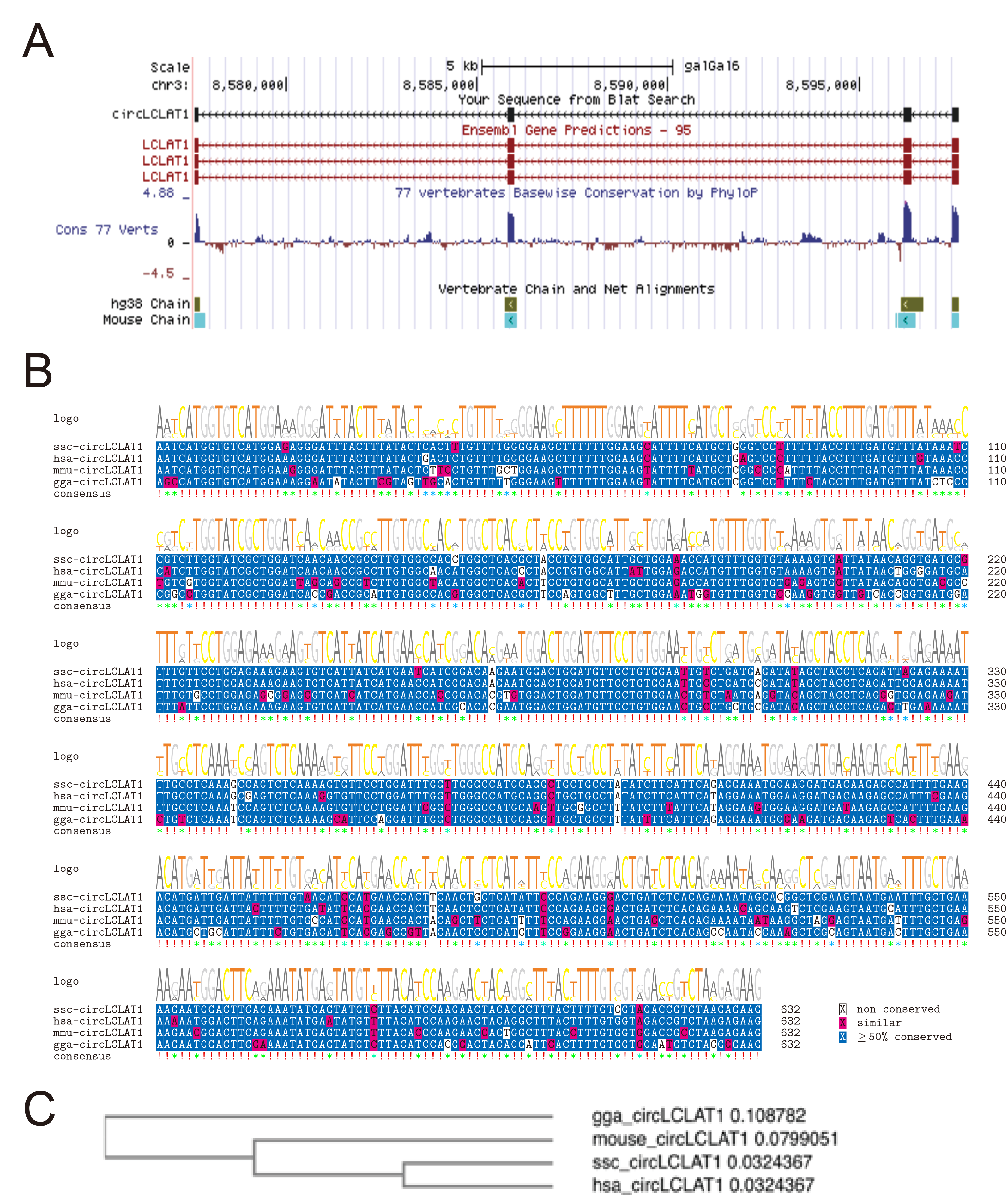

Supplement: Supplementary file 2 — Additional file 2 Figure S1. circLCLAT1 is a sequence-conservative circRNA. (A) Genome locate of chicken circLCLAT1. (B) Multiple sequence alignment analysis of circLCLAT1 among species. (C) The evolutionary tree of circLCLAT1 among species. [file 12864_2020_7000_MOESM2_ESM.tif]

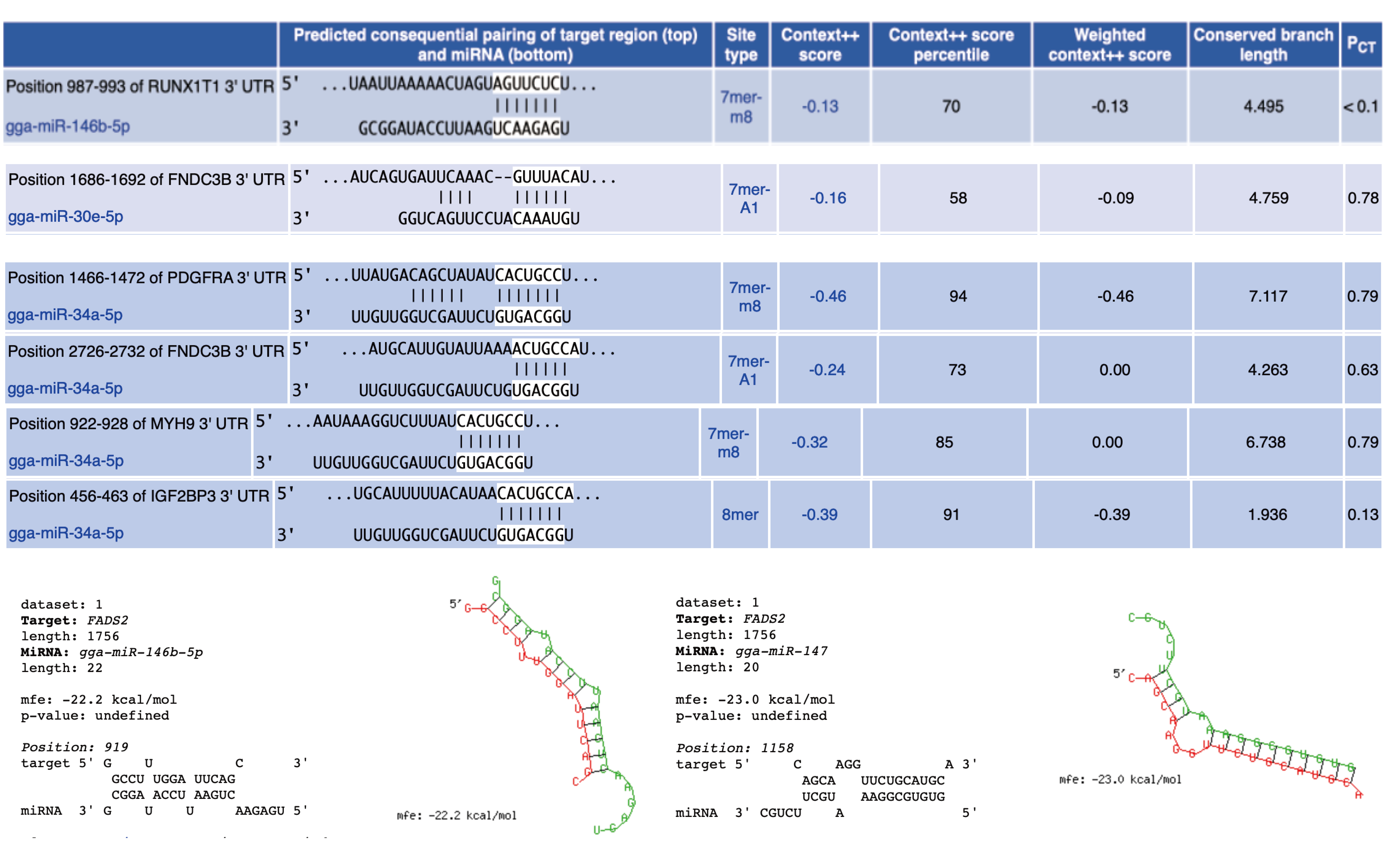

Supplement: Supplementary file 3 — Additional file 3 Figure S2. The binding sites of some upregulated miRNAs (eg. miR-146b-5p, miR-147, miR-34a-5p, miR-30e-5p) in the 3’UTRs of potential target genes. [file 12864_2020_7000_MOESM3_ESM.tif]
